# Protective Effect of *Phoenix dactylifera* L. Seeds against Paracetamol-Induced Hepatotoxicity in Rats: A Comparison with Vitamin C

**DOI:** 10.1155/2021/6618273

**Published:** 2021-07-06

**Authors:** Eimad Dine Tariq Bouhlali, Mgal Derouich, Abdelbassat Hmidani, Bouchra Bourkhis, Tarik Khouya, Younes Filali-Zegzouti, Chakib Alem

**Affiliations:** ^1^National Institute of Agronomic Research, Regional Center of Errachidia, Errachidia, Morocco; ^2^Biochemistry of Natural Products Team, Faculty of Sciences and Techniques, Moulay Ismail University, Errachidia, Morocco; ^3^Biology, Environment and Health Team, Faculty of Sciences and Techniques, Moulay Ismail University, Errachidia, Morocco; ^4^Faculty of Medicine and Pharmacy of Fes, Fes, Morocco

## Abstract

*Phoenix dactylifera* L. (date palm) seeds have been mentioned in the Moroccan pharmacopoeia as efficient remedies against a wide range of diseases including hepatic and gastrointestinal disorders and countless infections. The current work was performed to assess the phenolic profile and hepatoprotective potential of two date seed varieties, locally known as *Jihl* and *Majhoul*, aqueous extracts against paracetamol- (PCM-) driven liver toxicity in 42 Wistar rats. The polyphenol profile was built by means of an HPLC analysis. Hepatic damage was provoked by exposing rats to PCM at a dose of 1.5 g/kg once a week. Besides PCM, *Jihl* and *Majhoul* date seed extracts (200 and 400 mg/kg) were administered orally in a day-to-day routine. Our findings showed that among the examined polyphenol compounds, *p*-coumaric acid, quercetin, caffeic acid, and rutin were the most abundant phytochemicals. Date pits significantly (*p* < 0.001) stabilized the PCM-driven alterations in liver function parameters (AST, ALT, ALP, LDH, total protein, direct bilirubin, and total bilirubin). Moreover, *Phoenix dactylifera* pits enhanced considerably (*p* < 0.001) the activities of antioxidant enzymes (SOD, CAT, and GPx) as well as the level of reduced glutathione (GSH). The established hepatoprotective effect may be due to the date seeds antioxidant effect and their ability to trap free radicals. The main outcomes of the present study could validate the traditional use of these date seeds to manage various health conditions.

## 1. Introduction

The liver is a key site of detoxification and metabolization of drugs and xenobiotics in the human body [1]. Excessive or inaccurate use of drugs can lead to liver diseases, accounting for over half of all cases of acute liver failure [2]. PCM is a common analgesic and antipyretic drug which is regarded as a safe medication at therapeutic doses because it is mostly metabolized to pharmacologically inactive glucuronide and sulfate conjugates, with a minor fraction (5–10%) being oxidized to a reactive metabolite known as *N*-acetyl-*p*-benzoquinone imine (NAPQI) [2]. NAPQI, generated by hepatic cytochrome P-450 isoforms (CYP2E1 and CYP2A6) which are mainly responsible for PCM-induced hepatotoxicity, is efficiently detoxified through its binding to the sulfhydryl group of glutathione (GSH) and ultimately excreted in the urine as cysteine and mercapturic acid conjugates [3]. However, at high doses of PCM (more than 4 g/day), the sulfation and glucuronidation pathways become saturated, while oxidation increases, leading to an excessive production of NAPQI which depletes the hepatic GSH stores and causes oxidative stress [3]. These NAPQI entities covalently bind to cysteine groups on cellular proteins, especially mitochondrial proteins and ion channels causing the inhibition of energy production, ion imbalance, and cell death [3].

Current synthetic drugs used to treat liver disorders are ineffective and may have harmful side effects [4]. Thus, natural compounds derived from herbal plants have recently gained scientific interest as a new promising path to treat liver injuries.

In folk medicine, the powder of date palm (*Phoenix dactylifera* L.) seeds is used by several producing countries to treat various diseases including gastrointestinal disorders, diarrhoea, liver disorders, throat diseases, cancer, toothaches, diabetes, and pulmonary and numerous infections [5, 6]. In Moroccan traditional medicine, date seeds are implemented to treat jaundice and used as detoxifier. Our previous researches have proved that date seed extracts possess various pharmacologic activities, including antioxidant, anti-inflammatory, and hypolipidemic properties, which are linked to their high phenolic and flavonoid contents [7–9]. Previous studies have evidenced that date seeds possessed a high hepatoprotective potential on CCl_4_-treated rats [4, 10]. However, until now, no study has been conducted to assess the hepatoprotective effect of date seeds against PCM-induced liver damage as well as to evaluate the difference of efficacy that may exist between seeds from different varieties. Thus, the current study was designed to examine the phenolic profile of two date seed varieties locally known as *Jihl* and *Majhoul* grown in the southeast of Morocco and to inspect their effect on PCM-induced liver damage in Wistar rats in order to provide a scientific basis for their use in traditional medicine.

## 2. Materials and Methods

### 2.1. Preparation of Polyphenol-Rich Extracts

The rich phenolic extract was prepared according to the technique of Bouhlali et al. [11]. Concisely, 30 g of date seeds powder was extracted using 150 mL water, at 35°C for 12 h using an orbital shaker-incubator. The mixture was then sifted and the remainder was concentrated under reduced pressure at 40°C until total vaporization of solvent by means of a rotary evaporator. The final crude extracts were preserved at −20°C in dark glass flasks prior to analysis.

### 2.2. Identification and Quantification of Phenolic and Flavonoid Compounds

Phenolic acids and flavonoids of date seed varieties were determined using high-performance liquid chromatography (HPLC) as described by Bouhlali et al. [9], with a minor modification. One gram of date seed powder was first solubilized in 25 mL acidified methanol solution (1 N HCl/methanol/water, 1/80/19, v/v/v) and then ultrasonicated for 30 min. The mixture was centrifuged at 1000 rpm for 15 min and 2 mL of the supernatant was finally filtered via 0.45 *μ*m filter before its injection. Phenolic standards (comprising seven phenolic acids: caffeic, *p*-coumaric, chlorogenic, ferulic, gallic, vanillic, and syringic acids and three flavonoids: luteolin, quercetin, and rutin) were set at a stock solution of 100 *μ*g/mL and used to prepare calibration curves. Analytical separation was conducted on a Shimadzu HPLC system (Kyoto, Japan) equipped with an autosampler (SIL-20A), a dual pump (LC-20AB), a vacuum degasser (DGU-20A), a diode array detector (SPD-M10A), and a system controller (SCL-10A). The Restek C18 column (150 × 4.6 mm, 5 *μ*m particle size) (Bellefonte, USA) was used for polyphenol separation at 40°C. The binary mobile phase consisted of water-acetic acid (97 : 3, v/v) (eluent A) and acetonitrile (eluent B). Elution was performed at a flow rate of 1 mL/min with the following gradient outline: 0–5 min, 0–8% solution B (linear gradient); 5–25 min, 8–25% solution B (linear gradient); 25–30 min, 25% solution B (isocratic elution); and 30–50 min, 25–90% solution B (linear gradient). For starter, 20 microliters of each extract was injected and wavelengths of detection were set at 280, 320 and 350 nm. The compounds were identified based on their retention time and by spiking with phenolic standards under the same conditions, and their quantities were determined by means of a standard curve. The analysis was performed in triplicate, and the results were averaged and reported as milligrams per 100 g dried weight of date seeds.

### 2.3. Animals

Forty-two male Wistar rats (200–240 g) were used in the experiments. The animals were gotten from the animal facility of biology department (Faculty of Sciences and Techniques, Errachidia, Morocco). They were housed in plastic cages under a 12 h light/12 h dark cycle in a controlled temperature room (22 ± 2°C), with free access to food and water.

### 2.4. Experiment Design

Seven groups of six rats each were set up in a random manner:

- First group (normal): animals received distilled water orally for seven days
- Group II (PCM): rats were given distilled water orally and daily for six days; on the fifth day, rats received oral dose of PCM (1.5 g/kg)
- Group III (ascorbic acid): rats were gavaged daily with ascorbic acid (450 mg/kg) for six days; on the fifth day, rats received oral dose of PCM (1.5 g/kg)
- Groups IV and V (*Jihl* seed extract): rats were gavaged daily with *Jihl* seed extract at respective doses of 200 and 400 mg/kg for six days; on the fifth day, rats received oral dose of PCM (1.5 g/kg)
- Groups VI and VII (*Majhoul* seed extract): rats were gavaged daily with *Majhoul* seed extract at respective doses of 200 and 400 mg/kg for six days; on the fifth day, rats received oral dose of PCM (1.5 g/kg)

Ether solvent was used to anesthetize the rats following 24 h PCM treatment, sampling of blood was performed from the retro-orbital vein and the serum was centrifuged for 10 minutes at 2500 rpm and then subjected to biochemical analysis. After sacrificing the animals, their liver was taken out directly.

### 2.5. Preparation of Liver Homogenate

A portion of the median lobe of rat liver was washed with ice-cold at 0.9% NaCl. The homogenates were prepared at a ratio of 1 g sample of liver tissue in 10 mL ice-cold potassium phosphate buffer (1.25%; pH 7.0) using a glass-Teflon homogenizer. Each homogeneous solution was subjected to centrifugation for 20 min at 600 rpm and later on their supernatants were recovered and stored at −80°C until manipulation.

### 2.6. Determination of Hepatotoxicity Serum Indices

Serum AST (aspartate transaminase), ALT (alanine transaminase), ALP (alkaline phosphatase), LDH (lactate dehydrogenase), total protein, and direct and total bilirubin were assessed by means of assay kits from Randox Laboratories Ltd., UK, according to the company directions.

### 2.7. Determination of Oxidative Stress Markers in Liver Tissue

The superoxide dismutase (SOD), reduced glutathione (GSH), glutathione peroxidase (GPx), and catalase (CAT) activities were assessed, in the liver homogenate, according to the protocol provided by marketable supplies (Biodiagnostic, Giza, Egypt).

### 2.8. Statistical Analysis

The experimental results are expressed as mean ± standard deviation. Data were subjected to a one-way analysis of variance (ANOVA) implementing Statistical Package for the Social Sciences (SPSS, version 23) software. The control group (PCM alone treated group) and date seeds extracts groups were investigated by a post-hoc Dunnett's *t*-test. *p* < 0.05 was considered as statistically significant.

## 3. Results

### 3.1. Identification and Quantification of Phenolic and Flavonoid Compounds

**Figure 1:**
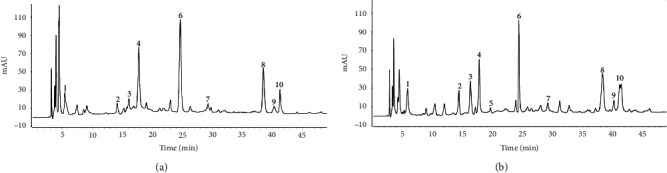
HPLC-DAD chromatograms of different date seed varieties. *Majhoul* seed (a). *Jihl* seed (b). Peak numbers: gallic acid (1); chlorogenic acid (2); vanillic acid (3); caffeic acid (4); syringic acid (5); *p*-coumaric acid (6); ferulic acid (7); rutin (8); luteolin (9); and quercetin (10).

Quantification of phenol-derived compounds was performed implementing the HPLC analysis. The main results are depicted in Table 1 and Figure 1. The examined date seed extracts showed substantial discordances (*p* < 0.05). The two varieties contained all the examined compounds except for *Jihl* seeds which did not contain syringic acid. The major phenolic compound was *p*-coumaric acid (107.03–119.96 mg/100 g DW), followed by caffeic acid (63.80–70.27 mg/100 g DW), gallic acid (16.37–18.42 mg/100 g DW), ferulic acid (9.18–11.40 mg/100 g DW), vanillic acid (12.60–20.42 mg/100 g DW), and chlorogenic acid (11.88–17.41 mg/100 g DW). The syringic acid was only found in the *Majhoul* date seed extract (3.87 mg/100 g DW). Regarding flavonoid compounds, rutin (54.11–58.56 mg/100 g DW) was found to be at higher concentration followed by quercetin (19.63–26.62 mg/100 g DW) and luteolin (9.01–12.08 mg/100 g DW). *Jihl* date seeds recorded the highest levels of *p*-coumaric, chlorogenic acid, vanillic acid, ferulic acid, gallic acid, syringic acid, quercetin, and luteolin, whereas *Majhoul* date seeds showed high levels of caffeic acid and rutin.

### 3.2. Effect of Date Seed Extracts and Ascorbic Acid on Serum Hepatospecific Markers

**Figure 2:**
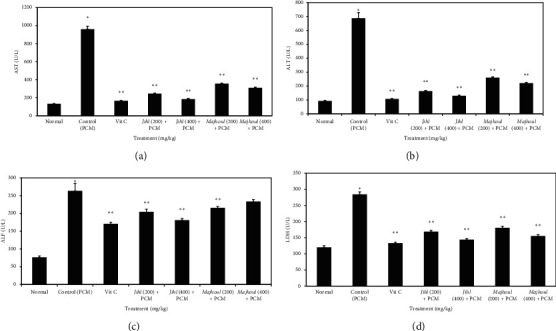
Effect of aqueous date seed extracts on PCM-driven changes in serum AST, ALT, ALP, and LDH activities. ALT: alanine aminotransferase, AST: aspartate aminotransferase, ALP: alkaline phosphatase, and LDH: lactate dehydrogenase. PCM: paracetamol (1500 mg/kg), Vit C: vitamin C (450 mg/kg). Values are means and standard deviation (*n* = 6 rats). The error bars represent the standard deviation of measurements in six samples (*n* = 6). ^*∗*^*p* < 0.001 (Dunnett's *t*-test) considerably discrepant compared to the normal group. ^*∗∗*^*p* < 0.001 (Dunnett's *t*-test) considerably discrepant compared to the control (PCM) group.

As depicted in Figure 2 and Table 2, the PCM group revealed a significant increase (*p* < 0.001) in serum levels of AST, ALT, ALP, LDH, and direct and total bilirubin along with a notable reduction in the total protein when compared to the normal control. However, as compared to the PCM-treated group, the administration of different date seed varieties extracts significantly reduced in a dose-dependent manner (*p* < 0.001) the levels of AST, ALT, ALP, LDH, and direct and total bilirubin along with remarkable elevation in the total protein. No considerable discrepancies (*p* < 0.001) were found between *Majhoul* date seed extract treated group at a dose of 400 mg/kg and negative control (PCM) group concerning ALP level. The establishment of normal serum parameters observed for *Jihl*, which was the most potent hepatoprotective date seed extract, was not as high as in ascorbic acid (200 mg/kg) treated rats.

### 3.3. Effect of Date Seed Extract and Ascorbic Acid on Liver Antioxidant Enzymes

**Figure 3:**
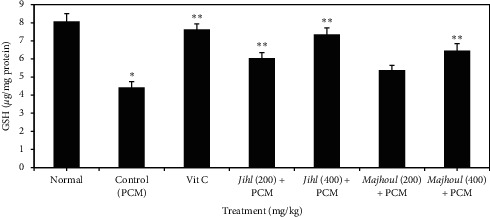
Effect of aqueous date seed extracts on hepatic GSH amount in rats with acute paracetamol (PCM) exposure. PCM: paracetamol (1500 mg/kg). Vit C: vitamin C (450 mg/kg). Values are means and standard deviation. The error bars signify the standard deviation of measurements in six samples (*n* = 6). ^*∗*^*p* < 0.001 (Dunnett's *t*-test) considerably discrepant compared to the normal group. ^*∗∗*^*p* < 0.001 (Dunnett's *t*-test) considerably discrepant compared to the control (PCM) group.

Compared to normal rats, PCM treatment significantly lowered (*p* < 0.001) activities of liver enzymatic antioxidants (SOD, CAT, and GPx) as well as nonenzymatic antioxidant (GSH) (Table 3 and Figure 3). Prefeeding with different date seed extracts considerably boosted these hepatic antioxidant enzymes compared to PCM-treated group. Similarly, pretreatment with ascorbic acid (450 mg/kg) elevated liver GSH level, SOD, CAT, and GPx activities significantly (*p* < 0.001). No considerable discrepancies (*p* < 0.001) were observed between *Majhoul* date seed extract (200 mg/kg) and PCM-received group. Among the studied date seed varieties, *Jihl* date seeds showed the highest improvement of liver enzymatic and nonenzymatic antioxidants.

## 4. Discussion

The current work was undertaken to assess the protective effect of date seed extracts against PCM-induced hepatotoxicity in Wistar rats in order to provide a scientific basis for their use in traditional medicine. PCM-driven liver injury is one of the most used experimental models to assess drug hepatoprotective [12]. In fact, the increased serum levels of all these biomarkers in PCM-treated rats compared to the control group confirmed the hepatic damage and indicated that the model had been successfully built. Hepatocyte function and integrity can be assessed by estimating the serum levels of various biomarkers such as ALT, AST, ALP, LDH, bilirubin (total and direct), and total protein, which are originally present in the cytoplasm [13]. These enzymes and molecules spill into the bloodstream during hepatopathy and act as markers of liver injury [13].

ALT predominantly found in liver unlike AST which is also abundantly present in other organs, namely, cardiac muscle and kidneys. For this reason, ALT is more specific than AST in detecting liver inflammation, through the parallel increase in ALT and AST levels [14]. Clinicians often use the AST/ALT ratio to distinguish between extrahepatic and hepatic damage, with an AST/ALT ratio of 2 : 1 indicating hepatic injury [15]. Accordingly, the AST/ALT ratio observed for the PCM-treated group may confirm the hepatic damage caused by this drug. Moreover, the elevated serum ALP level observed, in this study, could be attributable to defective hepatic excretion or increased ALP synthesis by hepatic parenchymal or duct cells in the presence of increasing biliary pressure as reported by Iyanda and Adeniyi [16]. Besides, the increased levels of bilirubin observed in PCM-treated group may be attributable to reduced hepatic clearance, indicating that it is a functional marker rather than a marker of cellular integrity as reflected by serum transaminase levels [14].

The reduction in total protein levels seen in PCM-treated rats suggests the destruction of many hepatic cells, which may result in a decrease in hepatic capacity to synthesis protein as the majority of plasma proteins are synthesized by hepatocytes [17].

Serum LDH levels were shown to be substantially greater following ischemic injury and PCM hepatotoxicity. However, when this elevation is accompanied by an increase in ALT and total bilirubin as found in this study, it becomes a valid indicator of liver damage [18].

Pretreatment with date seed extracts and ascorbic acid protected efficiently the rats against PCM-induced hepatic damage, as proven by the lowered ALT, AST, ALP, and LDH enzyme levels and raised total protein level as compared to PCM-treated group. Our results are consistent with those of Abdelaziz and Ali [10] who have found that co-administration of date seed extract to CCl_4_-treated rats restored the liver function parameters (AST, ALT, ALP, and albumin), lipid peroxidation (MDA), and activities of hepatic antioxidant enzymes that had been disrupted by CCl_4_ treatment. Moreover, in line with our finding, Al-Qarawi et al. [4] have found that date seed might act as prophylactic and cure against CCl_4_-induced hepatotoxicity in rats as a significant reduction in elevated AST, ALT, ALP, and bilirubin values was found in rats subjected to an intraperitoneal injection of CCl_4_ either before or after administration of date seed aqueous extracts.

The normalization of serum biomarkers by date seed extracts and ascorbic acid suggests that they may have provided protection against PCM toxicity by preserving the structural integrity of the hepatocellular membrane and, most likely, due to their membrane stabilizing activity which prevents intracellular enzymes leakage. Indeed, the same date seeds varieties have displayed a high and dose-dependent membrane stabilizing effect, especially *Jihl* date seeds which revealed the highest hepatoprotective effect [7].

Oxidative stress is an important mechanism that has been proposed to have a role in the development of PCM toxicity [3]. Accordingly, the glutathione, SOD, and CAT activities in the PCM intoxicated group were significantly lower compared to the control. The administration of date seed extracts improved considerably and dose dependently the enzymatic (SOD, CAT, and GPx) and nonenzymatic (GSH) antioxidant levels compared to the PCM alone treated animals. In fact, several reports have found that antioxidants supplementation may prevent PCM-induced hepatotoxicity [3]. Our previous preliminary phytochemical analysis revealed that date seeds contained significant amounts of total phenolics, flavonoids, condensed tannins, and strong antioxidant, anti-lipid peroxidation properties [8, 9]. Several studies have revealed that pretreatment with *p*-coumaric acid, caffeic acid, chlorogenic acid, gallic acid, syringic acid, vanillic acid, rutin, luteolin, or quercetin found in the analyzed date seeds for seven consecutive days prevented pathological increases of AST and ALT and improved liver antioxidant potency in rats subjected to PCM-induced hepatic damage by increasing SOD, GSH, and CAT activities [19–27]. On the other hand, among the CYP isoforms reported to be responsible for the NAPQI production, the in vitro tests revealed that luteolin and quercetin inhibited CYP 1A2 and CYP 3A4 [28], chlorogenic acid caused an inhibition of CYP 2E1 and CYP 1A2 enzymatic activities, but not CYP 3A4 [21], caffeic acid inhibited CYP 3A4 and CYP 2E1 [23], rutin was found to be a very potent inhibitor of CYP 3A4 [29], and ferulic acid downregulated the expression of CYP 2E1 [24]. As a result, the likelihood synergy between these compounds acting via discordant mechanisms on liver organic and functional aspects could justify the hepatoprotective effect of date seed extracts. Furthermore, the high amount of these compounds within *Jihl* date seeds may explain their most powerful hepatoprotective effect compared to *Majhoul* date seeds.

Importantly, the findings of the current work evidenced that date seeds aqueous extracts significantly and dose-dependently reduced the PCM-induced hepatotoxicity by enhancing hepatocyte antioxidant status.

## 5. Conclusion

Our finding demonstrated that aqueous date seed extracts were effective in preventing the deleterious effects of PCM administration. This was manifested by reducing serum biomarkers (ALT, LDH, AST, ALP, total protein, total bilirubin, and direct bilirubin) as well as enhanced enzymatic (SOD, CAT, and GPx) and nonenzymatic (GSH) antioxidant levels. *Jihl* date seeds recorded the greatest hepatoprotective effect when compared to the *Majhoul* date seeds. The extracts preventative efficacy might be attributed to their strong antioxidant capacity, membrane stabilizing action, and potential ability to suppress CYP, which is responsible for NAPQI formation, as well as their enhancement of liver enzymatic antioxidants. Our findings may support the traditional use of date seeds to treat to soothe jaundice and used as detoxifier.

## Figures and Tables

**Table 1:** Phenolic acids and flavonoid profiles determined by HPLC in two Moroccan date seed varieties extracts (mg/100 g DW).

	*Jihl* seeds	*Majhoul* seeds
*Phenolic acids*		
Caffeic acid	63.80 ± 1.08^a^	70.27 ± 1.61^b^
Chlorogenic acid	17.41 ± 0.73^c^	11.88 ± 0.61^d^
*p*-Coumaric acid	119.96 ± 1.34^e^	107.03 ± 1.97^f^
Ferulic acid	11.40 ± 0.65^g^	9.18 ± 0.71^h^
Gallic acid	18.42 ± 0.81^i^	16.37 ± 0.69^j^
Syringic acid	3.87 ± 0.54	ND
Vanillic acid	20.42 ± 0.92^k^	12.60 ± 0.71^m^

*Flavonoids*		
Luteolin	12.08 ± 0.74^n^	9.01 ± 0.82^p^
Quercetin	26.62 ± 0.86^q^	19.63 ± 0.71^r^
Rutin	54.11 ± 0.81^s^	58.56 ± 1.02^t^

Values are displayed as mean (*n* = 3) and standard error. ND : not determined. Means in the same line followed by different letters are significantly different from each other according to post hoc Bonferroni tests (*p* < 0.05).

**Table 2:** Effect of aqueous date seed extracts on serum AST, ALT, and ALP activities as well as total protein and direct and total bilirubin in rats with acute paracetamol (PCM) exposure.

Group	Treatment (mg/kg)	Total protein (mg/dL)	Direct bilirubin (mg/dL)	Total bilirubin (mg/dL)
Normal	Vehicle	7.88 ± 0.49	1.73 ± 0.22	5.80 ± 0.16
Control (PCM)	1500	4.48 ± 0.24^*∗*^	8.26 ± 0.20^*∗*^	29.11 ± 1.52^*∗*^
Ascorbic acid	450	7.12 ± 0.32^*∗∗*^	4.87 ± 0.29^*∗∗*^	13.75 ± 0.59^*∗∗*^

*Jihl* seed + PCM	400	6.81 ± 0.21^*∗∗*^	2.92 ± 0.23^*∗∗*^	7.96 ± 0.40^*∗∗*^
	200	6.37 ± 0.12^*∗∗*^	3.92 ± 0.32^*∗∗*^	10.32 ± 0.57^*∗∗*^

*Majhoul* seed + PCM	400	5.90 ± 0.16^*∗∗*^	5.64 ± 0.22^*∗∗*^	13.57 ± 0.46^*∗∗*^
	200	5.58 ± 0.16^*∗∗*^	6.10 ± 0.32^*∗∗*^	15.56 ± 0.51^*∗∗*^

Values are means and standard deviation (*n* = 6 rats). ^*∗*^*p* < 0.001 (Dunnett's *t*-test) considerably discrepant compared to the normal group. ^*∗∗*^*p* < 0.001 (Dunnett's *t*-test) considerably discrepant compared to the control (PCM) group.

**Table 3:** Effect of aqueous date seed extracts on hepatic antioxidant enzymes SOD, CAT, and GPx in rats with acute paracetamol (PCM) exposure.

Groups	Treatment (mg/kg)	SOD (U/mg protein)	CAT (U/mg protein)	GPx (U/mg protein)
Normal	Vehicle	22.86 ± 0.40	35.14 ± 1.11	22.86 ± 0.40
Paracetamol (PCM)	1500	9.25 ± 0.28^*∗*^	19.82 ± 1.15^*∗*^	9.25 ± 0.28^*∗*^
Vitamin C	450	20.65 ± 0.30^*∗∗*^	33.46 ± 0.67^*∗∗*^	20.65 ± 0.30^*∗∗*^
*Jihl* seed + PCM	400	23.44 ± 0.40^*∗∗*^	33.10 ± 0.83^*∗∗*^	23.44 ± 0.40^*∗∗*^
*Jihl* seed + PCM	200	20.97 ± 0.25^*∗∗*^	29.67 ± 0.95^*∗∗*^	20.97 ± 0.25^*∗∗*^
*Majhoul* seed + PCM	400	22.05 ± 0.32^*∗∗*^	31.03 ± 0.98^*∗∗*^	22.05 ± 0.32^*∗∗*^
*Majhoul* seed + PCM	200	19.61 ± 0.21^*∗∗*^	28.65 ± 0.90^*∗∗*^	19.61 ± 0.21^*∗∗*^

Values are expressed as means and standard deviation (*n* = 6 rats). ^*∗*^*p* < 0.001 (Dunnett's *t*-test) considerably discrepant compared to the normal group. ^*∗∗*^*p* < 0.001 (Dunnett's *t*-test) considerably discrepant compared to the control (PCM) group.

## Data Availability

All the data supporting the results are included within the article.
